# Altered Mitochondrial Dynamics and TBI Pathophysiology

**DOI:** 10.3389/fnsys.2016.00029

**Published:** 2016-03-30

**Authors:** Tara D. Fischer, Michael J. Hylin, Jing Zhao, Anthony N. Moore, M. Neal Waxham, Pramod K. Dash

**Affiliations:** ^1^Department of Neurobiology and Anatomy, McGovern Medical School, University of Texas Health Science Center at HoustonHouston, TX, USA; ^2^Department of Psychology, Southern Illinois UniversityCarbondale, IL, USA; ^3^Vivian L. Smith Department of Neurosurgery, McGovern Medical School, University of Texas Health Science Center at HoustonHouston, TX, USA

**Keywords:** Drp1, mitochondrial dynamics, neurodegeneration, neurogenesis, TBI

## Abstract

Mitochondrial function is intimately linked to cellular survival, growth, and death. Mitochondria not only generate ATP from oxidative phosphorylation, but also mediate intracellular calcium buffering, generation of reactive oxygen species (ROS), and apoptosis. Electron leakage from the electron transport chain, especially from damaged or depolarized mitochondria, can generate excess free radicals that damage cellular proteins, DNA, and lipids. Furthermore, mitochondrial damage releases pro-apoptotic factors to initiate cell death. Previous studies have reported that traumatic brain injury (TBI) reduces mitochondrial respiration, enhances production of ROS, and triggers apoptotic cell death, suggesting a prominent role of mitochondria in TBI pathophysiology. Mitochondria maintain cellular energy homeostasis and health via balanced processes of fusion and fission, continuously dividing and fusing to form an interconnected network throughout the cell. An imbalance of these processes, particularly an excess of fission, can be detrimental to mitochondrial function, causing decreased respiration, ROS production, and apoptosis. Mitochondrial fission is regulated by the cytosolic GTPase, dynamin-related protein 1 (Drp1), which translocates to the mitochondrial outer membrane (MOM) to initiate fission. Aberrant Drp1 activity has been linked to excessive mitochondrial fission and neurodegeneration. Measurement of Drp1 levels in purified hippocampal mitochondria showed an increase in TBI animals as compared to sham controls. Analysis of cryo-electron micrographs of these mitochondria also showed that TBI caused an initial increase in the length of hippocampal mitochondria at 24 h post-injury, followed by a significant decrease in length at 72 h. Post-TBI administration of Mitochondrial division inhibitor-1 (Mdivi-1), a pharmacological inhibitor of Drp1, prevented this decrease in mitochondria length. Mdivi-1 treatment also reduced the loss of newborn neurons in the hippocampus and improved novel object recognition (NOR) memory and context-specific fear memory. Taken together, our results show that TBI increases mitochondrial fission and that inhibition of fission improves hippocampal-dependent learning and memory, suggesting that strategies to reduce fission may have translational value after injury.

## Introduction

Mitochondria are at the crossroad between cellular health, survival, and death. Mitochondria not only provide cellular energy through ATP synthesis, but also play an important role in intracellular calcium buffering, reactive oxygen species (ROS) production, and apoptosis. A growing body of literature from both clinical and experimental brain injury research has shown that structural and functional damage of mitochondria is an early event after traumatic brain injury (TBI) that contributes to cell death and poor cognitive outcome (Vink et al., 1990; Okonkwo and Povlishock, 1999; Sullivan et al., 1999; Lifshitz et al., 2003, 2004; Singh et al., 2006; Cheng et al., 2012; Gajavelli et al., 2015). Decreased respiration and reduced ATP production in cortical and hippocampal mitochondria occurs within 24 h post-injury and can last up to 14 days in experimental models of TBI (Xiong et al., 1997; Lifshitz et al., 2003; Singh et al., 2006; Gilmer et al., 2009). Moreover, mitochondrial damage can result in the release of pro-apoptotic factors, such as cytochrome C, that activate cell death pathways and initiate apoptosis (Raghupathi et al., 2000; Brustovetsky et al., 2002). As neurons have high metabolic needs and do not store excess energy, continuous energy production and metabolic maintenance by functional mitochondria is critical for survival, supporting the premise that improving mitochondrial function can offer neuroprotection and improve cognition following TBI (Cheng et al., 2012; Gajavelli et al., 2015).

**Figure 1:**
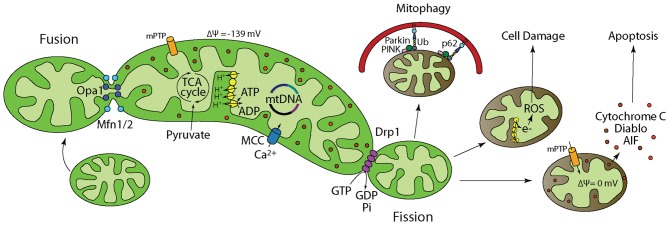
**Mitochondrial dynamics and function regulate cellular energy homeostasis, cell survival, growth, and death.** Inside of a cell, mitochondria are in a dynamic flux, continuously undergoing fusion and fission to meet cellular energy needs. (1) Fusion is regulated by Optic Atrophy 1 (Opa1) and Mitofusin1/2 (Mfn1/2) and allows for functional complementation and repair of damaged mitochondria. (2) Pyruvate from glycolysis is transported into the mitochondria where pyruvate dehydrogenase catalyzes the decarboxylation of Pyruvate to form Acetyl CoA. Acetyl CoA then enters the TCA cycle. (3) Electromotive force generated by the electron transport chain allows ATP synthesis by the F1-Fo ATP synthase. (4) Mitochondria buffer excess intracellular calcium. When calcium buffering capacity is compromised as a result of mitochondrial damage/depolarization, calcium accumulates in the cytosol and activates degradative enzymes such as calpain and phospholipases that break down cellular proteins and membrane leading to death. (5) Fission is regulated by dynamin-related protein 1 (Drp1) and allows for mitochondria that cannot be repaired to be isolated followed by degradation through mitophagy. Fission is also important for subcellular distribution and transportation of mitochondria based on energy needs in certain areas of the cell. (6) Electrons leaked from the electron transport chain interact with molecular oxygen to generate reactive oxygen species (ROS) that not only damage mitochondrial membrane, mitochondrial DNA (mtDNA), and proteins, but also their cellular counter parts. Neurons have limited defense against oxidative damage and are highly vulnerable to ROS. (7) Mitochondria play a prominent role in apoptotic cell death. Damaged/depolarized mitochondria release cytochrome c and apoptosis inducing factor (AIF) that trigger cell death by activating caspases.

Mitochondria are dynamic organelles that continuously undergo fusion and fission to form a highly interconnected network throughout the cell (Bereiter-Hahn and Vöth, 1994; Chan, 2006; van der Bliek et al., 2013). These balanced processes alter mitochondrial morphology and allow mitochondria to efficiently respond to cellular energy needs (Bereiter-Hahn and Vöth, 1994; Chan, 2006; Westermann, 2012; van der Bliek et al., 2013). Fusion allows for an increase in cristae density and maximization of ATP production during high metabolic activity and stress (Westermann, 2012; Youle and van der Bliek, 2012). In contrast, fission allows for proliferation and transportation of mitochondria to areas with energy demands, in addition to segregation of damaged mitochondria from the network for subsequent degradation through mitophagy (Youle and van der Bliek, 2012; Otera et al., 2013). An imbalance between fusion and fission, particularly an excess of fission, can be detrimental for energy homeostasis and has been implicated in neurodegenerative diseases (Detmer and Chan, 2007; Knott and Bossy-Wetzel, 2008; Knott et al., 2008; Archer, 2013; Burté et al., 2015). More specifically, excessive fission can lead to reduced mitochondrial respiration and ATP production, increased ROS generation, and release of apoptogenic factors, changes similar to those seen after TBI (Figure 1; Rintoul et al., 2003; Chen et al., 2005; Cribbs and Strack, 2007; Detmer and Chan, 2007; Yu et al., 2008a; Chen and Chan, 2009; Costa et al., 2010; Jahani-Asl et al., 2011; Jheng et al., 2012). Dynamin-related protein 1 (Drp1) is a key regulator of mitochondrial fission, through its interactions with the mitochondrial outer membrane (MOM; van der Bliek et al., 2013). Prior to a fission event, Drp1 translocates to the MOM where it self-assembles and forms an oligomeric structure around the mitochondrion. Hydrolysis of Drp1-bound GTP then drives the subsequent mitochondrial membrane division. Mitochondrial division inhibitor-1 (Mdivi-1) is an allosteric inhibitor of Drp1 that inhibits its oligomeric assembly thereby reducing its GTP binding affinity (Cassidy-Stone et al., 2008). Mdivi-1 has been shown to reduce cell death by attenuating mitochondrial fission in yeast, and in animals models (Cassidy-Stone et al., 2008; Jahani-Asl et al., 2011; Grohm et al., 2012; Rappold et al., 2014; Zhao et al., 2014). Recently, a study has reported that Mdivi-1 reduces cortical cell loss and improves spatial memory after TBI in mice (Wu et al., 2016). However, it is unknown if TBI alters Drp1 translocation to the MOM and mitochondrial dynamics.

In the present study, we measured Drp1 levels in purified mitochondria from the hippocampi of brain injured animals and found increased translocation. CryoEM analysis of images of mitochondria also showed a reduction in their lengths 72 h after TBI that was blocked by Mdivi-1 treatment. Further, we show that injured animals receiving Mdivi-1 have reduced loss of newborn neurons in the hippocampus and improved novel object recognition (NOR) memory and contextual fear memory. These results indicate that TBI enhances mitochondrial fission which contributes to poor cognitive outcome. Thus, strategies aimed at reducing mitochondrial fission can reduce pathology and may have translational value to treat TBI.

## Materials and Methods

### Materials

Adult, male Sprague Dawley rats (300–400 g) were purchased from Charles River Laboratories (Wilmington, MA, USA). Mdivi-1 was purchased from Tocris Bioscience (Bristol, UK). Antibodies for Drp1 (westerns) and Doublecortin (DCX) were purchased from Cell Signaling Technology (Danvers, MA, USA) whereas Drp1 (immuno-gold), TOMM20 and GAPDH antibodies were purchased from Abcam (Cambridge, MA, USA).

### Controlled Cortical Impact (CCI) Injury

All procedures were approved by the Institutional Animal Care and Use Committee (IACUC) prior to initiating these studies. An electromagnetic Controlled Cortical Impact (CCI) device was used for experimental TBI as previously described (Dixon et al., 1991; Brody et al., 2007; Hoskison et al., 2009). Animals were anesthetized using 5% isofluorane with a 1:1 O_2_/N_2_O mixture and then mounted on a stereotaxic frame with anesthesia maintained with 2.5% isofluorane in 1:1 O_2_/air. Bilateral 6 mm craniectomies were produced midway between the bregma and lambda (offset 0.5 mm from midline) and a single impact (2.5 mm deformation) was given at a velocity of 5 m/s to the right parietal cortex. Sham-operated animals received all surgical procedures described above excluding the craniectomies and impact. Recovery of pain reflexes and restoration of the righting response were recorded immediately after surgery to ascertain consistency in the injury. For mitochondrial isolation experiments, animals underwent all described procedures, except the TBI was delivered using an Impact One^TM^ Leica Biosystems CCI device with a deformation of 2.8 mm.

### Drug Preparation and Administration

Mdivi-1 was dissolved in DMSO to a concentration of 25 mg/ml, after which it was diluted to a working concentration of 1.5 mg/ml. For testing the influence of Mdivi-1 on cognitive function and histopathology after TBI, injured animals were randomly assigned to either vehicle (40% DMSO) or 3 mg/kg Mdivi-1 groups. This dosage of Mdivi-1 was based on previous studies that demonstrated neuroprotection with Mdivi-1 treatment (Grohm et al., 2012). For behavioral testing, injured animals were randomly assigned to two groups and i.p. injected at 30 min, 24 h, and 48 h post-injury with Mdivi-1 (3 mg/kg) or an equivalent volume of vehicle. For immunohistochemistry, animals were injected at 30 min post-injury and again at 8 h post-injury.

### Mitochondrial Isolation

To isolate mitochondria from brain tissues, Percoll density gradient centrifugation was used as previously described (Sims and Anderson, 2008). Hippocampi from two animals per experimental group were pooled together to increase the quantity of starting material. Hippocampi from sham animals and ipsilateral (to the injury) hippocampi from injured animals were rapidly removed. Tissue was homogenized in ice-cold isolation buffer (100 mM Tris pH 7.4, 10 mM EDTA, 12% Percoll solution, 1 mM Sodium Fluoride, 1 mM Sodium Molybdate, 100 nM Okadaic Acid, 1 mM PMSF and 10 μg/ml leupeptin). The tissue was then homogenized (4 strokes) in a Dounce homogenizer using the loose pestle followed by 8 strokes using the tight pestle. A small fraction of each homogenate was removed for determination of protein content. The remaining homogenate was then layered onto a discontinuous Percoll gradient (26% and 40% Percoll) and centrifuged for 8 min (30,700 g at 4°C). The enriched mitochondrial fraction was removed from the 26/40% interface, transferred to individual centrifuge tubes, and diluted (1:4) with isolation buffer. Fractions were then pelletized by centrifugation (16,700 g at 4°C) for 10 min. The supernatant was discarded and the samples were either immediately prepared for electron microscopy analysis or frozen and stored at −80°C. Samples underwent one freeze-thaw cycle prior to western analysis.

### Western Blotting

Protein concentrations of the total homogenate and the mitochondrial fractions (*n* = 3 samples/group, each sample was pooled from two animals) were determined using a Bicinchoninic Acid (BCA) protein assay (Thermo Scientific^TM^ Protein Biology) with BSA as the standard. Equal amounts of protein for each sample were resolved using a SDS-PAGE and transferred to Immobilon-P membranes (Millipore, Bedford, MA, USA). Membranes were blocked overnight at 4°C with SuperBlock^™^ (TBS; ThermoFisher Scientific, Grand Island, NY, USA) and then incubated in primary antibody solutions (Drp1, 1:1000; TOMM20, 0.5 μg/ml; GAPDH, 1 μg/ml) for 3 h at room temperature. The membrane was then washed and incubated with species-specific, horseradish peroxidase-conjugated, secondary antibodies for 1 h. Immunoreactivity was detected using SuperSignal^TM^ West Pico chemiluminescent substrate (ThermoFisher Scientific; Grand Island, NY, USA) and exposure to Kodak XAR5 film (Rochester, NY, USA). The relative optical density of each band was analyzed using ImageJ (NIH).

### Transmission Electron Microscopy and Gold Immunolabeling

Freshly isolated mitochondria from rat hippocampi were applied to freshly glow-discharged (30 s) carbon-coated copper grids, blotted, and then fixed with 4% paraformaldehyde for 15 min on a chilled plate. Excess sample was blotted away and grids were blocked sample-side down on a 50 μL drop of blocking buffer (5% BSA, 1× HBS). Grids were then floated on a drop of primary antibody (Drp1, 0.02 mg/ml or TOMM20, 0.01 mg/ml) for 30 min and washed before incubation in 12-nm gold-conjugated secondary antibody. The grids were washed and stained in methylamine vanadate (Nanoprodes, Nanovan), blotted, and air dried. CCD images of isolated mitochondria were taken on a JEOL1400 transmission electron microscope running at 120 kV with a Gatan Orius SC1000 camera.

### Cryo-Electron Microscopy and Mitochondrial Length Measurements

Freshly isolated mitochondria from rat hippocampi (*n* = 1 sample/group, each sample was pooled from two animals) were immediately applied to freshly glow-discharged (30 s) 2/2 Quantifoil on 200 mesh copper grids. After 30 s, excess buffer was blotted and the sample was immediately plunged into ethane cooled to liquid N_2_ temperature. Cryo-preserved grids were stored in liquid N_2_ until use. Cryo-electron microscopy was performed on a FEI Polara G2 equipped with a Gatan K2 Summit direct electron detector. Multiple areas of the grid were chosen at random and 8 × 8 montages were collected at 4700× in low dose/photon counting mode using SerialEM. To quantify the length of mitochondria, individual montages were displayed in IMOD and a line along the long axis of each mitochondrion was drawn and stored in a model for each montage. Lengths were extracted for 200 mitochondria from each model table, imported into Excel and the data displayed by separating the lengths into 500 nm bins. To remove potential bias, the person collecting the primary data and the person quantifying the length of individual mitochondria were both blinded as to the sample identities.

### Immunohistochemistry

For immunohistochemistry, groups of animals were injured, and were randomly assigned to receive either vehicle or 3 mg/kg Mdivi-1 (*n* = 5/group). Animals were killed at 24 h post-CCI by an overdose of sodium pentobarbital and exsanguination with ice cold phosphate-buffered-saline (PBS) followed by 4% paraformaldehyde. Brains were collected and post-fixed in 4% PFA for 24 h before step-wise cryopreservation in sucrose (15% to 30% in PBS) at 4°C. Brains were cut into 40 μm-thick coronal sections using a cryostat. Tissue sections containing the dorsal hippocampus were selected, permeabilized in PBS containing 0.25% Triton-X100, blocked for 1 h in 2.5% normal goat serum, then incubated in primary antibody solutions overnight. After extensive washing, tissues were incubated in a species-specific biotinylated secondary antibody followed by incubation in ABC solution (Vectastain ABC kit, Vector Laboratories, Burlingame, CA, USA). Tissues were then developed in 3,3′-diaminobenzidine (DAB) solution, washed and mounted on gelatin-subbed slides and allowed to dry overnight. Tissues were dehydrated in an alcohol series, delipidated in xylenes, and coverslipped with Permount. DCX-positive cells were then counted and the dentate gyrus measured (mm) using a Zeiss *Axiovert* brightfield microscope. Measurements were performed by an experimenter blind experimenter to sample identity.

### Behavioral Tasks

Separate groups of animals were used for behavioral analysis. Animals were injured and randomly assigned to receive either vehicle (*n* = 6) or 3 mg/kg Mdivi-1 (*n* = 8). A group of sham-injured animals was prepared and used as baseline controls (*n* = 8). All behavioral tasks were carried out by an experimenter that was unaware of the treatment groups.

#### Novel Object Recognition (NOR)

The NOR task is a hippocampal/entorhinal cortex-dependent learning and memory task that assesses novel vs. familiar object recognition (Ennaceur and Delacour, 1988). Animals were placed in the testing chamber (100 × 100 cm box) and allowed to habituate for 10 min per day for 2 days. On day three, two identical objects were placed in the box and the animal was allowed to explore the objects for 10 min. Twenty-four hours later, a new object of the same color and size, but different shape replaced one of the objects and the animal was allowed to again explore the objects for 10 min. The time spent exploring each object was recorded by a blind experimenter on both the familiarization and testing days. The difference in percent time exploring the novel vs. the familiar object [(time spent exploring object/total time exploring) × 100] on the testing day is used as a measure of recognition memory.

#### Contextual Fear Conditioning

The one-trial contextual fear conditioning (FC) procedure was adapted from Wiltgen et al. (2006) and Drew et al. (2010). The training chamber included only visual contextual cues, but no auditory stimuli (i.e., a tone). The protocol did not include an acclimation exposure before training to ensure no prior association with the context. On the first day, the animal was placed in the training chamber and allowed to familiarize itself with the context for 150 s before receiving a mild foot shock (2 s, 0.7 mA). Thirty seconds later, the animals were removed from the training chamber and returned to their home cage. Twenty-four hours later, the animal was placed back in the training chamber for 3 min (without shock). Freezing behavior (defined as the absence of movement except that needed for respiration) was recorded for both the training and the testing day every 2 s within the 3 min time period by an experimenter who was unaware of the treatment groups. The percent time the animal remained frozen was calculated and used as an index of fear memory.

### Statistical Analysis

For mitochondrial morphology, data were analyzed using the Kruskal-Wallis Analysis of Ranks with Tukey’s method for *post hoc* comparisons and the Mann-Whitney Rank Sum Test. For changes of immunoreactivity across time in western analysis, data were analyzed using a one-way analysis of variance (ANOVA), with a Bonferroni method for *post hoc* analysis. For NOR, Student’s *t*-tests were used to determine any significant difference in time spent between each object pair during familiarization and testing. For contextual FC, a Student’s *t*-test was used to determine differences between vehicle-treated and Mdivi-1 treated freezing behavior on the testing day. Results were considered significant at *p* < 0.05. Data are presented as the mean ± standard error of the mean (SEM).

## Results

### TBI Increases Drp1 Association with Mitochondria

**Figure 2:**
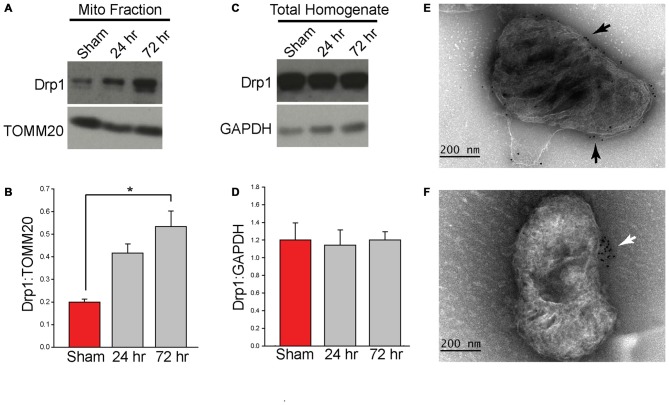
**Controlled cortical impact (CCI) injury increases the level of Drp1 in isolated hippocampal mitochondrial fractions.** Animals were injured by CCI and mitochondria were isolated from injured hippocampal tissues at 24 h and 72 h post-injury. **(A)** Representative western blots of isolated ipsilateral hippocampal mitochondrial fractions from sham and injured (24 and 72 h) animals. **(B)** Relative immunoreactivity of each band was quantified and Drp1 optical density values were normalized to TOMM20. Drp1 levels increase marginally at 24 h and significantly at 72 h (*p* = 0.007) post-injury. **(C)** Representative western blots from ipsilateral hippocampal homogenate from sham and injured (24 and 72 h) animals. **(D)** Immunoreactivity was quantified and optical density values for Drp1 were normalized to GAPDH. Results indicate no Drp1 level changes in the ipsilateral hippocampus. Immuno-gold labeling was also performed using a primary antibody for TOMM20, a mitochondrial outer membrane (MOM) translocase, and Drp1 with a gold-conjugated secondary antibody. **(E)** Representative image of TOMM20 immuno-gold labeling on the MOM. Gold particles indicated by black arrows. **(F)** Representative image of Drp1 immuno-gold labeling on a mitochondrion from the injured hippocampus. Gold labeling for Drp1 was found to be associated with the isolated mitochondria and associated with the MOM (indicated by white arrow). Data are presented as the mean ± standard error of the mean (SEM). *p < 0.05.

Translocation of Drp1 to the MOM is critical for fission (Chang and Blackstone, 2007; Cereghetti et al., 2008; Santel and Frank, 2008). Employing protein extracts from purified mitochondrial (*n* = 3 samples/group, each sample was pooled from two animals), western blots were performed to determine if TBI alters mitochondrial-associated Drp1 levels. Figure 2A shows representative western blot images indicating an immunoreactive band at approximately 80 kDa [corresponding to the expected migration of Drp1; Cell Signaling Technology (Danvers, MA, USA)] that appears to increase at both 24 h and 72 h after injury. TOMM20 immunoreactivity was used to normalize loading. Quantification of Drp1 immunoreactivity (Figure 2B; *n* = 3/group) revealed that Drp1 levels are increased in mitochondrial fractions after TBI (*F*_(2,6)_ = 13.059, *p* = 0.007), reaching statistical significance by 72 h post-injury (*p* = 0.007). To determine if TBI alters total Drp1 levels (both cytosolic and mitochondria-associated), hippocampal homogenates from sham and injured animals (*n* = 3/group) were also compared by western blotting. Drp1 optical density was normalized with the optical density for the cytoplasmic protein, GAPDH. Results presented in Figures 2C,D indicate no significant changes in total hippocampal Drp1 levels as a result of CCI injury. In order to assess if the translocated Drp1 is present on the MOM, we performed immuno-gold labeling and electron microscopy analysis. We first performed immuno-gold labeling using antibodies for the outer membrane translocase (TOMM20). A representative electron micrograph is shown in Figure 2E indicates positive TOMM20 staining with localization of gold particles (black dots) on the outer surface of the mitochondrion. Figure 2F shows a representative picture for Drp1 immuno-gold labeling (Abcam; Cambridge, MA, USA) of a mitochondrion from the injured hippocampus. Gold signal (black dots) can be seen clustered on the outer membrane of this mitochondrion.

### TBI Decreases Mitochondrial Length that is Blocked by Mdivi-1 Treatment

**Figure 3:**
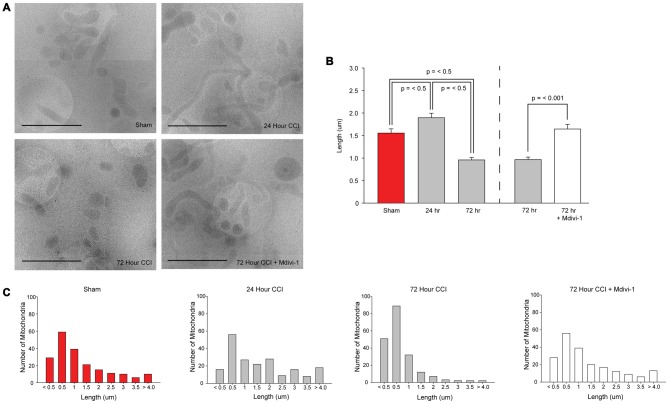
**CCI injury decreases mitochondrial length in the hippocampus that is blocked by Mitochondrial division inhibitor-1 (Mdivi-1). (A)** Representative cryoEM micrographs of isolated hippocampal mitochondria from sham, 24 h post-CCI, 72 h post-CCI, and 72 h post-CCI + Mdivi-1. **(B)** Summary results for average mitochondrial length for each group. Please note, the results for the 72 h injured group are presented twice to represent the statistical comparisons to assess mitochondria length over time after injury, and to assess the effect of Mdivi-1 at 72 h. **(C)** Population distributions of hippocampal mitochondrial lengths from sham, 24 h post-CCI, 72 h post-CCI, and 72 h post-CCI + Mdivi-1 animals. Lengths were binned (bin sizes of 500 nm) and the number of mitochondria per bin are shown. *X*-axis labels indicate the lower bin value for each bin range. Data are presented as the mean ± SEM.

To determine if TBI alters mitochondrial length, mitochondria were isolated from sham and injured hippocampi. CryoEM micrographs were captured and used for measuring mitochondrial length (200 mitochondria/sample; each sample was pooled from two animals; *n* = 1 sample/group). Figure 3A shows representative cryoEM micrographs of mitochondria isolated from sham and injured hippocampal tissues. Mitochondria of different lengths can be seen in all groups. However, longer mitochondria are more present in the 24 h sample. In contrast, smaller mitochondria are more present in the 72 h sample. The summary results shown in Figure 3B indicate that TBI significantly alters mitochondrial length (*H* = 71.413, *p* = < 0.001) with a significant increase in average length observed at 24 h post-injury (diff. of ranks = 28.922, *p* = < 0.05), followed by a significant decrease at 72 h post-injury (diff. of ranks = 18.514, *p* < 0.05), compared to sham injured controls. In order to examine if an inhibitor of Drp1 can attenuate the reduction in mitochondrial length detected 72 h after TBI, a group of injured animals was administered 3 mg/kg (i.p) Mdivi-1 starting at 30 min post-injury, and again at 24, 48, and 72 h post-injury. Mitochondria were isolated from injured hippocampi as described in the “Materials and Methods” Section. The representative cryoEM micrographs shown in Figure 3A indicates longer mitochondria in Mdivi-1 treated animals as compared to untreated injured animals. Quantification of mitochondrial lengths (from 200 mitochondria; *n* = 1 sample pooled from two animals) revealed that Mdivi-1 significantly increased mitochondrial length at 72 h post-injury (*U* = 13.097, *p* = < 0.001; Figure 3B).

To examine differences in distribution of mitochondrial size, lengths were binned (bin sizes of 500 nm) and the number of mitochondria per bin was recorded. Figure 3C shows that in sham animals, 63.5% of the mitochondria are less than 1.5 μm in length. Twenty-four hours after TBI, there is a modest decrease in the number of mitochondria less than 1.5 μm (49.5%) with a shift in the distribution in favor of mitochondria with lengths between 2 μm and 4 μm. By 72 h post-injury, 86% of mitochondria are between 200 nm and 1.5 μm, with very few mitochondria longer than 2 μm. In the Mdivi-1 treated group, the distribution of mitochondria was similar to that seen in sham controls, with 61.5% of mitochondria less than 1.5 μm in length.

### Mdivi-1 Treatment Reduces Newborn Neuronal Loss Following TBI

**Figure 4:**
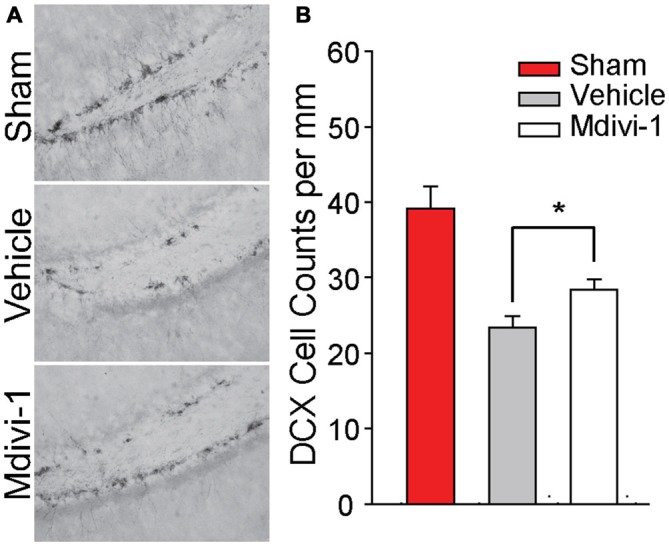
**Mdivi-1 treatment reduces loss of newborn neurons in the dentate gyrus of the hippocampus after CCI injury. (A)** Representative photomicrographs of Doublecortin (DCX) positive staining in the dentate gyrus of the ipsilateral hippocampus in a sham and 24 h post-injury animals (vehicle-treated and Mdivi-1 treated). **(B)** Quantification of DCX+ cells in the ipsilateral and contralateral dentate gyrus for each group revealed that animals treated with Mdivi-1 had increased DCX+ cells in the ipsilateral hippocampus compared to vehicle-treated controls, suggesting reduced loss of newly born neurons. Data are presented as the mean ± SEM. *p < 0.05.

Recently, it has been reported that Mdivi-1 treatment reduces cortical cell loss following TBI (Wu et al., 2016). In addition to cortical cell loss, both experimental and clinical TBI causes loss of hippocampal neurons, including DCX-positive newborn neurons in the dentate gyrus (Hall et al., 2005; Gao et al., 2008; Yu et al., 2008b; Christidi et al., 2011). We therefore examined if post-injury Mdivi-1 treatment can attenuate the loss of these newborn neurons. Groups of animals were injured, and were randomly assigned to receive either vehicle or 3 mg/kg Mdivi-1 (*n* = 5/group) 30 min post-injury. A second injection was administered 8 h later. Animals were euthanized 24 h post-injury for examination of DCX-positive neurons. Figure 4A shows representative photomicrographs of DCX-positive cells in the subgranular zone of the ipsilateral hippocampus from sham and injured (vehicle-treated and Mdivi-1 treated) animals. Consistent with previous studies, CCI injury caused a marked reduction of DCX-positive neurons in the ipsilateral hippocampus (*t*_(8)_ = 4.817, *p* = 0.001), compared to sham animals, with no significant changes observed on the contralateral side. Mdivi-1 treatment significantly attenuated the loss of DCX-positive cells in the ipsilateral hippocampus after injury (*t*_(8)_ = −2.381, *p* = 0.044; Figure 4B) as compared to injured animals treated with vehicle.

### Mdivi-1 Treatment Improves Recognition Memory and Contextual Fear Memory

**Figure 5:**
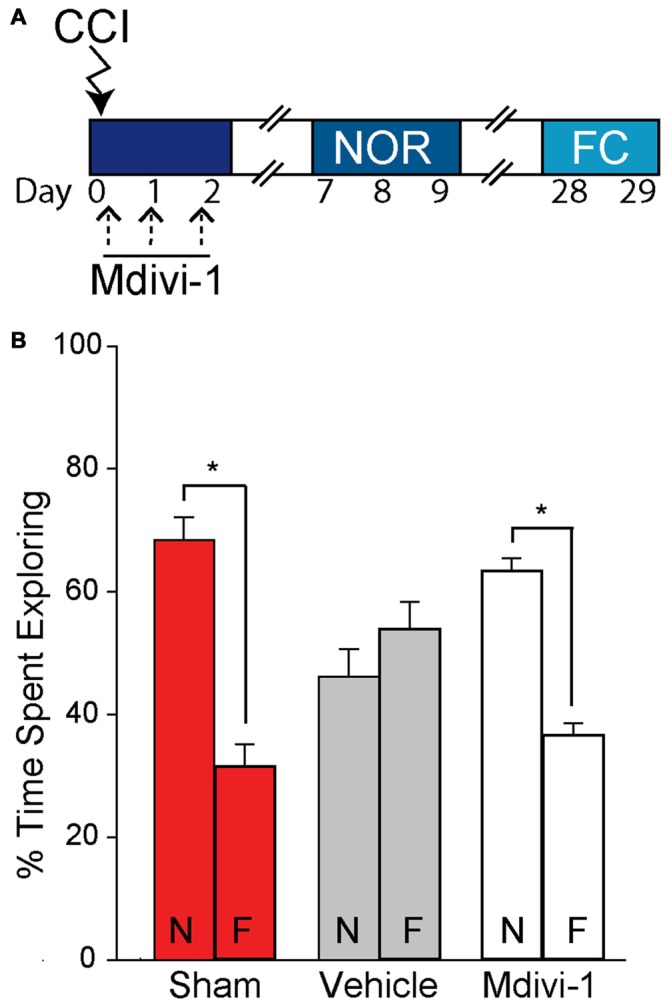
**Post-CCI mdivi-1 treatment rescues recognition memory after CCI injury.** CCI animals were treated with either 3.0 mg/kg of Mdivi-1 (*n* = 8) or vehicle (*n* = 6) starting 30 min post-injury, and subsequently at 24 and 48 h post-injury. **(A)** Representative timeline for Mdivi-1 treatment and behavioral experiments. Rats were familiarized with two identical objects during the training period and percent of time spent exploring each object was quantified to control for any pre-existing biases. No significant biases to either object were observed. **(B)** Twenty-four hours later, one of the objects was replaced by a novel object and percent of time spent exploring both the novel (N) and familiar (F) objects were recorded. Mdivi-1 treated animals demonstrated improved recognition memory in the Novel object recognition (NOR) task indicated by a significant increase in exploration of the novel object vs. the familiar object. Data are presented as the mean ± SEM. *p < 0.05. CCI, controlled cortical impact; FC, fear conditioning; NOR, novel object recognition.

As we observed that Mdivi-1 inhibits changes in mitochondrial length in the injured hippocampus and reduces the loss of newborn neurons after TBI, we questioned if this would result in improved learning and memory performance on the hippocampal-dependent NOR and context-specific fear memory tasks (Hernández-Rabaza et al., 2009; Drew et al., 2010; Denny et al., 2012; Kheirbek et al., 2012; Nakashiba et al., 2012; Suárez-Pereira et al., 2015). Sham (*n* = 8) and injured animals (vehicle-treated, *n* = 6; Mdivi-1 treated, *n* = 8) were used for this experiment according to the timeline shown in Figure 5A. During the familiarization phase of the NOR task all three groups equally explored both objects, suggesting no pre-existing biases or asymmetries. Long-term memory was tested 24 h later by replacing one of the familiar objects with a novel one. Sham animals spent significantly more time exploring the new object (Figure 5B; *t*_(14)_ = 7.270, *p* = < 0.001), indicating intact recognition memory. In contrast, injured animals receiving vehicle-treatment had impaired recognition memory, spending equivalent times exploring both the familiar and the novel object (*t*_(10)_ = −1.225, *p* = 0.249). Interestingly, Mdivi-1 treated injured animals show a significant preference for the novel object vs. the familiar object (*t*_(14)_ = 9.408, *p* = < 0.001; Figure 5B), indicating preserved recognition memory.

**Figure 6:**
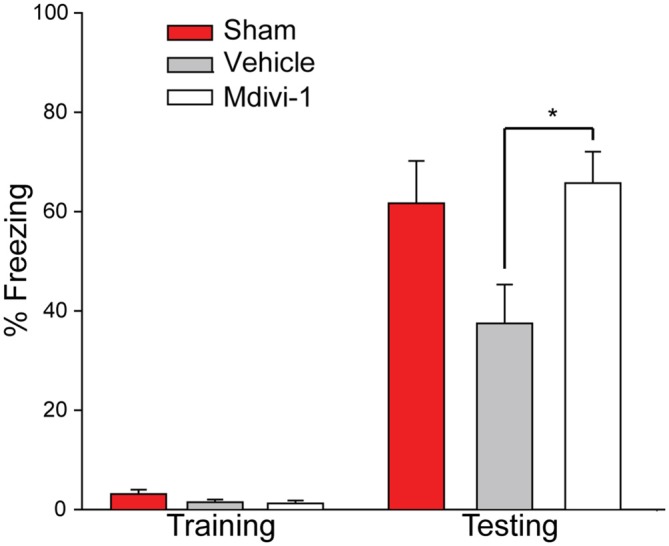
**Post-CCI mdivi-1 treatment rescues contextual memory after CCI injury.** CCI animals were treated with either 3.0 mg/kg of Mdivi-1 (*n* = 8) or vehicle (*n* = 6) starting 30 min post-injury, and subsequently at 24 and 48 h post-injury. Four weeks post-injury, rats were exposed to the FC training chamber, before receiving a mild foot shock (2 s, 7.0 mV). Prior to foot shock, animal in all three groups explored the context equally (training). Twenty-four hours later (one-trial) the rats were replaced back into the training chamber and their freezing behavior was recorded for a total of 3 min. Rats treated with Mdivi-1 demonstrated improved contextual fear memory in a one-trial context-specific FC task indicated by a significant increase in freezing behavior compared to vehicle-treated controls. Data are presented as the mean ± SEM. *p < 0.05.

The same animals were then tested 28 days post-injury on a one-trial context-specific FC task. Figure 6 shows that prior to delivery of a mild foot shock all animals were mobile and showed minimal freezing behavior in the training context. Twenty-four hours later, the memory for the training context was tested. Sham animals froze approximately 60% of the observation time. By comparison to the sham group, injured animals with vehicle treatment showed reduced freezing behavior. On the other hand, Mdivi-1 treated group froze at a level comparable to shams and significantly more than the vehicle-treated controls (*t*_(12)_ = −2.839, *p* = 0.015), indicating improved contextual fear memory.

## Discussion

Growing evidence in the literature indicates that mitochondria exist in a dynamic flux, undergoing continuous fission and fusion to meet cellular energy needs and an imbalance of these processes can be detrimental to cell survival (Rintoul et al., 2003; Chen et al., 2005; Cribbs and Strack, 2007; Detmer and Chan, 2007; Knott and Bossy-Wetzel, 2008; Knott et al., 2008; Yu et al., 2008a; Chen and Chan, 2009; Costa et al., 2010; Jahani-Asl et al., 2011; Jheng et al., 2012; Archer, 2013; Burté et al., 2015). In the present study, we investigated whether TBI alters mitochondrial dynamics. The results of this study reveal four key findings: (1) TBI reduces mitochondrial length 72 h after injury that is blocked by Mdivi-1, a pharmacological inhibitor of Drp1; (2) TBI does not alter total hippocampal Drp1, but rather increases the translocation of Drp1 to the mitochondria to initiate fission; (3) Mdivi-1 attenuates TBI-triggered death of newborn neurons; and (4) post-injury treatment with Mdivi-1 improves long-term memory in hippocampal-dependent tasks, namely NOR memory and context-specific fear memory. Our findings, along with recently published data, indicate that excessive mitochondrial fission may contribute to hippocampal neuronal death and neurocognitive impairments after TBI (Wu et al., 2016).

The dynamic processes of fusion and fission maintain metabolic homeostasis and allow mitochondria to efficiently respond to fluctuating energy needs (Bereiter-Hahn and Vöth, 1994; Chan, 2006; Westermann, 2012; van der Bliek et al., 2013). Mitochondrial fusion is regulated by large GTPases associated with the inner (Optic Atrophy 1, Opa1) and outer mitochondrial membranes (Mitofusins 1/2, Mfn1/2; Figure 1). Fusion promotes mixing of contents, thereby allowing for functional complementation of mitochondrial DNA (mtDNA) needed for repair of damaged mitochondria (Westermann, 2012; Youle and van der Bliek, 2012). Mitochondria also undergo hyperfusion in states of high energy demand to increase their cristae density to maximize ATP production (Westermann, 2012; Youle and van der Bliek, 2012). Mitochondrial fission is primarily regulated by the GTPase Drp1 and also serves a number of cellular functions (Bereiter-Hahn and Vöth, 1994; Chan, 2006; Westermann, 2012; Youle and van der Bliek, 2012; Otera et al., 2013; van der Bliek et al., 2013). By subdividing the network, mitochondria can proliferate and be transported to areas that require metabolic activity (Westermann, 2012; Youle and van der Bliek, 2012). Additionally, when mitochondria are damaged, fission allows for segregation from the network and subsequent degradation of the damaged portion of the mitochondrion through mitophagy (Youle and van der Bliek, 2012; Otera et al., 2013). Excessive fission events can cause small, fragmented mitochondria and have been implicated in mechanisms of neuronal death and dysfunction in many neurodegenerative conditions including Parkinson’s disease and Alzheimer’s disease (Knott and Bossy-Wetzel, 2008; Knott et al., 2008). Results of the current study show that length of hippocampal mitochondria is significantly decreased by 3 days post-injury, a finding consistent with excessive fission. Additionally, administration of the Drp1 inhibitor Mdivi-1 blocked TBI-induced decreases in mitochondrial length. In addition to a significant reduction in mitochondria length at 72 h post-injury, we observed a transient, but significant, increase in mitochondrial length at 24 h post-injury. An increase in mitochondrial length may indicate an acute period of increased mitochondrial fusion. Both clinical and experimental studies have shown there is an acute period of increased glucose utilization after TBI (Giza and Hovda, 2001; Prins et al., 2013; Gajavelli et al., 2015). As mitochondrial fusion has been shown to increase ATP production, the acute increase in mitochondrial length we observed may be consistent with increased glucose utilization after TBI. Alternatively, as increased fusion has been shown to rescue dysfunctional mitochondria through complementation of contents with healthy mitochondria, an acute increase in fusion after TBI may be indicative of such a mechanism as well (Westermann, 2012; Youle and van der Bliek, 2012). Future investigations are required to further characterize changes in the balance of fusion and fission and the contribution of these mechanisms to TBI pathology.

Approximately 97% of cellular Drp1 is cytosolic. In response to stimuli, Drp1 translocates to, and interacts with, the MOM protein Fis1 to initiate fission (Smirnova et al., 2001; van der Bliek et al., 2013). Consistent with this, the Drp1 immuno-gold labeling results show gold particles association with the MOM (Figure 2E). After translocation to the MOM, Drp1 self-assembles and forms an oligomeric structure around the mitochondrion. Hydrolysis of bound GTP then drives the subsequent membrane division. Dysregulation of Drp1 function has been linked to aberrant mitochondrial fission causing mitochondrial dysfunction and cell damage (Rintoul et al., 2003; Chen et al., 2005; Cribbs and Strack, 2007; Detmer and Chan, 2007; Yu et al., 2008a; Chen and Chan, 2009; Costa et al., 2010; Jahani-Asl et al., 2011; Jheng et al., 2012). Dysregulation of Drp1 can result from: (1) changes in Drp1 protein levels (e.g., transcription/translation); (2) changes in activity (e.g., post-translational modifications/phosphorylation); and/or (3) changes in translocation to the MOM (Frank et al., 2001; Reddy et al., 2011; Wang et al., 2011; Manczak and Reddy, 2012; Zhao et al., 2013). Our results reveal that although total Drp1 levels in hippocampal homogenates do not change after injury, mitochondrial association of Drp1 significantly increases 72 h after injury (Figure 2), indicating an increase in translocation from the cytosol. These results are consistent with the decrease in mitochondrial size also observed at 72 h after injury, indicating an increase in mitochondrial fission. Drp1 is highly responsive to environmental signaling and appears to be an important link in communicating changes in energy demands and allowing for appropriate changes in mitochondrial dynamics (Detmer and Chan, 2007; Santel and Frank, 2008; Otera et al., 2013). A number of intracellular signaling pathways have been shown to regulate mitochondrial fission by modifying Drp1, including phosphorylation, ubiquitination, sumolyation, and nitrosylation (Chang and Blackstone, 2007; Taguchi et al., 2007; Cereghetti et al., 2008; Knott et al., 2008; Santel and Frank, 2008; Kanamaru et al., 2012). However, whether these modifications of Drp1 occur after TBI are yet to be determined.

Recently, a study has shown that administration of Mdivi-1 decreases cortical cell loss and improves performance in the Morris water maze task after TBI (Wu et al., 2016). In addition to causing the loss of mature neurons, TBI causes the death of newborn neurons, with previous studies showing that approximately 50% of DCX-positive newborn neurons die within 24 h to 3 days of the injury (Hall et al., 2005; Gao et al., 2008; Yu et al., 2008b). Consistent with the neuroprotective effect of Mdivi-1, a significant protection of DCX-positive newborn neurons in the dentate gyrus of the hippocampus was observed after Mdivi-1 treatment. These results suggest that inhibiting mitochondrial fission may improve the survivability of newborn cells after TBI. This protection was associated with improved performance in behavioral tasks known to be influenced by treatments which impair ongoing neurogenesis (one-trial context-specific fear memory, Figure 6; Hernández-Rabaza et al., 2009; Drew et al., 2010; Denny et al., 2012; Kheirbek et al., 2012; Nakashiba et al., 2012; Suárez-Pereira et al., 2015). Although this improvement is consistent with the protection of newborn neurons, we cannot rule out the possibility that adult neurons protected by Mdivi-1 treatment could have contributed to the improved memory we observed.

While results of the current study indicate that TBI causes excessive mitochondrial fission, and that strategies to reduce fission can offer neuroprotection and improve outcome, a number of limitations exist that constrain interpretation of the results: (1) the morphological results were obtained from isolated hippocampal mitochondria. It is possible that the morphology of isolated mitochondria may not reflect the morphology of mitochondria *in vivo*; (2) as mitochondrial isolation was carried out from the entire hippocampus, the cell-type or regional specificity of fission events cannot be determined; (3) although Mdivi-1 has been shown to be a specific inhibitor of Drp1 *in vitro*, the *in vivo* specificity of this chemical can be questioned (Cassidy-Stone et al., 2008). Additionally, only one dosage of Mdivi-1 (3 mg/kg) was used in this study and whether higher or lower doses would yield similar results is yet to be determined. A dose response curve would be needed to determine the optimal dose for improvements in pathological and cognitive outcomes after injury.

## Summary

As mitochondrial dynamics play an integral role in cellular health, its dysregulation may affect neuronal survival and plasticity. The current results reveal that TBI causes a decrease in mitochondrial size and increased Drp1 translocation to mitochondria, indicating an increase in fission events. Inhibition of Drp1 with Mdivi-1 treatment restores mitochondrial length, reduces loss of newborn hippocampal neurons, and improves hippocampal-dependent learning and memory after injury. Overall, these results indicate a role for Drp1 and excessive mitochondrial fission in neuronal death and cognitive dysfunction after TBI. By investigating pathological changes in mitochondrial fission, these studies provide an innovative perspective on mechanisms of metabolic dysfunction and may lead to novel mitochondrial-targeted therapeutic approaches to improve outcome after brain injury.

## Author Contributions

TDF, MJH, JZ, ANM, MNW, and PKD have made substantial contributions to the design, acquisition, analysis, and interpretation of the original research described in this manuscript. All have played a role in the drafting and revision of the manuscript, and have approved of the final version. All authors agree to be accountable for all aspects of the work and ensure that questions related to the accuracy or integrity of any part of the work were appropriately investigated and resolved.

## Conflict of Interest Statement

The authors declare that the research was conducted in the absence of any commercial or financial relationships that could be construed as a potential conflict of interest.

## References

[B1] Archer S. (2013). Mitochondrial dynamics–mitochondrial fission and fusion in human diseases. N. Engl. J. Med. 369, 2236–2251. 10.1056/NEJMra1215233 24304053

[B2] Bereiter-Hahn J. Vöth M. (1994). Dynamics of mitochondria in living cells: shape changes, dislocations, fusion and fission of mitochondria. Microsc. Res. Tech. 27, 198–219. 10.1002/jemt.1070270303 8204911

[B3] Brody D. L. Mac Donald C. Kessens C. C. Yuede C. Parsadanian M. Spinner M.. (2007). Electromagnetic controlled cortical impact device for precise, graded experimental traumatic brain injury. J. Neurotrauma 24, 657–673. 10.1089/neu.2006.0011 17439349 PMC2435168

[B4] Brustovetsky N. Brustovetsky T. Jemmerson R. Dubinsky J. (2002). Calcium-induced cytochrome c release from CNS mitochondria is associated with the permeability transition and rupture of the outer membrane. J. Neurochem. 80, 207–218. 10.1046/j.0022-3042.2001.00671.x 11902111

[B5] Burté F. Carelli V. Chinnery P. F. Yu-Wai-Man P. (2015). Disturbed mitochondrial dynamics and neurodegenerative disorders. Nat. Rev. Neurol. 11, 11–24. 10.1038/nrneurol.2014.228 25486875

[B6] Cassidy-Stone A. Chipuk J. E. Ingermann E. Song C. Yoo C. Kuwana T.. (2008). Chemical inhibition of the mitochondrial division dynamin reveals its role in Bax/Bak-dependent mitochondrial outer membrane permeabilization. Dev. Cell 14, 193–204. 10.1016/j.devcel.2007.11.019 18267088 PMC2267902

[B7] Cereghetti G. M. Stangherlin A. Martins de Brito O. Chang C. R. Blackstone C. Bernardi P.. (2008). Dephosphorylation by calcineurin regulates translocation of Drp1 to mitochondria. Proc. Natl. Acad. Sci. U S A 105, 15803–15808. 10.1073/pnas.0808249105 18838687 PMC2572940

[B8] Chan D. (2006). Mitochondrial fusion and fission in mammals. Ann. Rev. Cell Dev. Biol. 22, 79–99. 10.1146/annurev.cellbio.22.010305.104638 16704336

[B9] Chang C. R. Blackstone C. (2007). Cyclic AMP-dependent protein kinase phosphorylation of Drp1 regulates its GTPase activity and mitochondrial morphology. J. Biol. Chem. 282, 21583–21587. 10.1074/jbc.c700083200 17553808

[B11] Chen H. Chan D. C. (2009). Mitochondrial dynamics-fusion, fission, movement and mitophagy-in neurodegenerative diseases. Hum. Mol. Genet. 18, R169–R176. 10.1093/hmg/ddp326 19808793 PMC2758711

[B10] Chen H. Chomyn A. Chan D. (2005). Disruption of fusion results in mitochondrial heterogeneity and dysfunction. J. Biol. Chem. 280, 26185–26192. 10.1074/jbc.m503062200 15899901

[B12] Cheng G. Kong R. Zhang L. Zhang J. (2012). Mitochondria in traumatic brain injury and mitochondrial-targeted multipotential therapeutic strategies. Br. J. Pharmacol. 167, 699–719. 10.1111/j.1476-5381.2012.02025.x 23003569 PMC3575772

[B13] Christidi F. Bigler E. D. McCauley S. R. Schnelle K. P. Merkley T. L. Mors M. B.. (2011). Diffusion tensor imaging of the perforant pathway zone and its relation to memory function in patients with severe traumatic brain injury. J. Neurotrauma 28, 711–725. 10.1089/neu.2010.1644 21381986

[B14] Costa V. Giacomello M. Hudec R. Lopreiato R. Ermak G. Lim D.. (2010). Mitochondrial fission and cristae disruption increase the response of cell models of Huntington’s disease to apoptotic stimuli. EMBO Mol. Med. 2, 490–503. 10.1002/emmm.201000102 21069748 PMC3044888

[B15] Cribbs J. T. Strack S. (2007). Reversible phosphorylation of Drp1 by cyclic AMP-dependent protein kinase and calcineurin regulates mitochondrial fission and cell death. EMBO Rep. 8, 939–944. 10.1038/sj.embor.7401062 17721437 PMC2002551

[B16] Denny C. A. Burghardt N. S. Schachter D. M. Hen R. Drew M. (2012). 4- to 6-week-old adult-born hippocampal neurons influence novelty-evoked exploration and contextual fear conditioning. Hippocampus 22, 1188–1201. 10.1002/hipo.20964 21739523 PMC3193906

[B17] Detmer S. A. Chan D. (2007). Functions and dysfunctions of mitochondrial dynamics. Nat. Rev. Mol. Cell Biol. 8, 870–879. 10.1038/nrm2275 17928812

[B18] Dixon C. E. Clifton G. L. Lighthall J. W. Yaghmai A. A. Hayes R. (1991). A controlled cortical impact model of traumatic brain injury in the rat. J. Neurosci. Methods 39, 253–262. 10.1016/0165-0270(91)90104-8 1787745

[B19] Drew M. Denny C. Hen R. (2010). Arrest of adult hippocampal neurogenesis in mice impairs single-but not multiple-trial contextual fear conditioning. Behav. Neurosci. 124, 446–454. 10.1037/a0020081 20695644 PMC2925248

[B20] Ennaceur A. Delacour J. (1988). A new one-trial test for neurobiological studies of memory in rats. 1: behavioral data. Behav. Brain Res. 31, 47–59. 10.1016/0166-4328(88)90157-x 3228475

[B21] Frank S. Gaume B. Bergmann-Leitner E. S. Leitner W. W. Robert E. G. Catez F.. (2001). The role of dynamin-related protein 1, a mediator of mitochondrial fission, in apoptosis. Dev. Cell 1, 515–525. 10.1016/s1534-5807(01)00055-7 11703942

[B22] Gajavelli S. Sinha V. K. Mazzeo A. T. Spurlock M. S. Lee S. W. Ahmed A. I.. (2015). Evidence to support mitochondrial neuroprotection, in severe traumatic brain injury. J. Bioenerg. Biomembr. 47, 133–148. 10.1007/s10863-014-9589-1 25358440

[B23] Gao X. Deng-Bryant Y. Cho W. Carrico K. M. Hall E. D. Chen J. (2008). Selective death of newborn neurons in hippocampal dentate gyrus following moderate experimental traumatic brain injury. J. Neurosci. Res. 86, 2258–2270. 10.1002/jnr.21677 18381764 PMC3757515

[B24] Gilmer L. K. Roberts K. N. Joy K. Sullivan P. G. Scheff S. (2009). Early mitochondrial dysfunction after cortical contusion injury. J. Neurotrauma 26, 1271–1280. 10.1089/neu.2008.0857 19637966 PMC2850255

[B25] Giza C. C. Hovda D. A. (2001). The neurometabolic cascade of concussion. J. Athl. Train. 36, 228–235. 12937489 PMC155411

[B26] Grohm J. Kim S. W. Mamrak U. Tobaben S. Cassidy-Stone A. Nunnari J.. (2012). Inhibition of Drp1 provides neuroprotection *in vitro* and *in vivo*. Cell Death Differ. 19, 1446–1458. 10.1038/cdd.2012.18 22388349 PMC3422469

[B27] Hall E. D. Sullivan P. G. Gibson T. R. Pavel K. M. Thompson B. M. Scheff S. W. (2005). Spatial and temporal characteristics of neurodegeneration after controlled cortical impact in mice: more than a focal brain injury. J. Neurotrauma 22, 252–265. 10.1089/neu.2005.22.252 15716631

[B28] Hernández-Rabaza V. Llorens-Martín M. Velázquez-Sánchez C. Ferragud A. Arcusa A. Gumus H. G.. (2009). Inhibition of adult hippocampal neurogenesis disrupts contextual learning but spares spatial working memory, long-term conditional rule retention and spatial reversal. Neuroscience 159, 59–68. 10.1016/j.neuroscience.2008.11.054 19138728

[B29] Hoskison M. M. Moore A. N. Hu B. Orsi S. Kobori N. Dash P. (2009). Persistent working memory dysfunction following traumatic brain injury: evidence for a time-dependent mechanism. Neuroscience 159, 483–491. 10.1016/j.neuroscience.2008.12.050 19167462 PMC4264540

[B30] Jahani-Asl A. Pilon-Larose K. McLaurin J. G. Park D. S. McBride H. M. Slack R. (2011). The mitochondrial inner membrane GTPase, optic atrophy 1 (Opa1), restores mitochondrial morphology and promotes neuronal survival following excitotoxicity. J. Biol. Chem. 286, 4772–4782. 10.1074/jbc.M110.167155 21041314 PMC3039390

[B31] Jheng H.-F. Tsai P.-J. Guo S.-M. Kuo L.-H. Chang C.-S. Su I.-J.. (2012). Mitochondrial fission contributes to mitochondrial dysfunction and insulin resistance in skeletal muscle. Mol. Cell. Biol. 32, 309–319. 10.1128/MCB.05603-11 22083962 PMC3255771

[B32] Kanamaru Y. Sekine S. Ichijo H. Takeda K. (2012). The phosphorylation-dependent regulation ofmitochondrial proteins in stress responses. J. Signal Transduct. 2012:931215. 10.1155/2012/931215 22848813 PMC3403084

[B33] Kheirbek M. A. Tannenholz L. Hen R. (2012). NR2B-dependent plasticity of adult-born granule cells is necessary for context discrimination. J. Neurosci. 32, 8696–8702. 10.1523/JNEUROSCI.1692-12.2012 22723709 PMC3388607

[B34] Knott A. B. Bossy-Wetzel E. (2008). Impairing the mitochondrial fission and fusion balance: a new mechanism of neurodegeneration. Ann. N Y Acad. Sci. 1147, 283–292. 10.1196/annals.1427.030 19076450 PMC2605288

[B35] Knott A. B. Perkins G. Schwarzenbacher R. Bossy-Wetzel E. (2008). Mitochondrial fragmentation in neurodegeneration. Nat. Rev. Neurosci. 9, 505–518. 10.1038/nrn2417 18568013 PMC2711514

[B36] Lifshitz J. Friberg H. Neumar R. W. Raghupathi R. Welsh F. A. Janmey P.. (2003). Structural and functional damage sustained by mitochondria after traumatic brain injury in the rat: evidence for differentially sensitive populations in the cortex and hippocampus. J. Cereb. Blood Flow Metab. 23, 219–231. 10.1097/00004647-200302000-00009 12571453

[B37] Lifshitz J. Sullivan P. G. Hovda D. A. Wieloch T. McIntosh T. (2004). Mitochondrial damage and dysfunction in traumatic brain injury. Mitochondrion 4, 705–713. 10.1016/j.mito.2004.07.021 16120426

[B38] Manczak M. Reddy P. (2012). Abnormal interaction between the mitochondrial fission protein Drp1 and hyperphosphorylated tau in Alzheimer’s disease neurons: implications for mitochondrial dysfunction and neuronal damage. Hum. Mol. Genet. 21, 2538–2547. 10.1093/hmg/dds072 22367970 PMC3349426

[B39] Nakashiba T. Cushman J. D. Pelkey K. A. Renaudineau S. Buhl D. L. McHugh T. J.. (2012). Young dentate granule cells mediate pattern separation, whereas old granule cells facilitate pattern completion. Cell 149, 188–201. 10.1016/j.cell.2012.01.046 22365813 PMC3319279

[B40] Okonkwo D. O. Povlishock J. (1999). An intrathecal bolus of cyclosporin A before injury preserves mitochondrial integrity and attenuates axonal disruption in traumatic brain injury. J. Cereb. Blood Flow Metab. 19, 443–451. 10.1097/00004647-199904000-00010 10197514

[B41] Otera H. Ishihara N. Mihara K. (2013). New insights into the function and regulation of mitochondrial fission. Biochim. Biophys. Acta 1833, 1256–1268. 10.1016/j.bbamcr.2013.02.002 23434681

[B42] Prins M. Greco T. Alexander D. Giza C. (2013). The pathophysiology of traumatic brain injury at a glance. Dis. Model. Mech. 6, 1307–1315. 10.1242/dmm.011585 24046353 PMC3820255

[B43] Raghupathi R. Graham D. I. McIntosh T. (2000). Apoptosis after traumatic brain injury. J. Neurotrauma 17, 927–938. 10.1089/neu.2000.17.927 11063058

[B44] Rappold P. M. Cui M. Grima J. C. Fac R. C. de Mesy-Bently K. L. Chen L.. (2014). Drp1 inhibition attenuates neurotoxicity and dopamine release deficits *in vivo*. Nat. Commun. 5:5244. 10.1038/ncomms6244 25370169 PMC4223875

[B45] Reddy P. H. Reddy T. P. Mannczak M. Calkins M. J. Shirendeb U. Mao P. (2011). Dynamin-related protein 1 and mitochondrial fragmentation in neurodegenerative diseases. Brain Res. Rev. 67, 103–118. 10.1016/j.brainresrev.2010.11.004 21145355 PMC3061980

[B46] Rintoul G. L. Filiano A. J. Brocard J. B. Kree G. L. Reynolds I. (2003). Glutamate decreases mitochondrial size and movement in primary forebrain neurons. J. Neurosci. 23, 7881–7888. 12944518 10.1523/JNEUROSCI.23-21-07881.2003 PMC6740596

[B47] Santel A. Frank S. (2008). Shaping mitochondria: the complex posttranslational regulation of the mitochondrial fission protein DRP1. IUBMB Life 60, 448–455. 10.1002/iub.71 18465792

[B48] Sims N. R. Anderson M. (2008). Isolation of mitochondria from rat brain using Percoll density gradient centrifugation. Nat. Protoc. 3, 1228–1239. 10.1038/nprot.2008.105 18600228

[B49] Singh I. N. Sullivan P. G. Deng Y. Mbye L. Hall E. (2006). Time course of post-traumatic mitochondrial oxidative damage and dysfunction in a mouse model of focal traumatic brain injury: implications for neuroprotective therapy. J. Cereb. Blood Flow Metab. 26, 1407–1418. 10.1038/sj.jcbfm.9600297 16538231

[B50] Smirnova E. Griparic L. Shurland D. L. van der Bliek A. (2001). Dynamin-related protein Drp1 is required for mitochondrial division in Mammalian cells. Mol. Biol. Cell 12, 2245–2256. 10.1091/mbc.12.8.2245 11514614 PMC58592

[B51] Suárez-Pereira I. Canals S. Carrión A. (2015). Adult newborn neurons are involved in learning acquisition and long-term memory formation: the distinct demands on temporal neurogenesis of different cognitive tasks. Hippocampus 25, 51–61. 10.1002/hipo.22349 25139443

[B52] Sullivan P. G. Thompson M. B. Scheff S. W. (1999). Cyclosporin A attenuates acute mitochondrial dysfunction following traumatic brain injury. Exp. Neurol. 160, 226–234. 10.1006/exnr.1999.7197 10630207

[B53] Taguchi N. Ishihara N. Jofuku A. OKa T. Mihara K. (2007). Mitotic phosphorylation of dynamic-related GTPase Drp1 participates in mitochondrial fission. J. Biol. Chem. 282, 11521–11529. 10.1074/jbc.m607279200 17301055

[B54] van der Bliek A. M. Shen Q. Kawajiri S. (2013). Mechanisms of mitochondrial fission and fusion. Cold Spring Harb. Prospect. Biol. 5:a011072. 10.1101/cshperspect.a011072 23732471 PMC3660830

[B55] Vink R. Head V. A. Rogers P. J. McIntosh T. K. Faden A. (1990). Mitochondrial metabolism following traumatic brain injury in rats. J. Neurotrauma 7, 21–27. 10.1089/neu.1990.7.21 2342116

[B56] Wang H. Song P. Du L. Tian W. Yue W. Liu M.. (2011). Parkin ubiquitinates Drp1 for proteasome-dependent degradation: implication of dysregulated mitochondrial dynamics in Parkinson disease. J. Biol. Chem. 286, 11649–11658. 10.1074/jbc.M110.144238 21292769 PMC3064217

[B57] Westermann B. (2012). Bioenergetic role of mitochondrial fusion and fission. Biochim. Biophys. Acta 1817, 1833–1838. 10.1016/j.bbabio.2012.02.033 22409868

[B58] Wiltgen B. J. Sanders M. J. Anagnostara S. G. Sage J. R. Fanselow M. S. (2006). Context fear learning in the absence of the hippocampus. J. Neurosci. 26, 5484–5491. 10.1523/jneurosci.2685-05.2006 16707800 PMC6675287

[B59] Wu Q. Xia S.-X. Li Q.-Q. Gao Y. Shen X. Ma L.. (2016). Mitochondrial division inhibitor 1 (Mdivi-1) offers neuroprotection through diminishing cell death and improving functional outcome in a mouse model of traumatic brain injury. Brain Res. 1630, 134–143. 10.1016/j.brainres.2015.11.016 26596858

[B60] Xiong Y. Gu Q. Peterson P. L. Muizelaar J. P. Lee C. (1997). Mitochondrial dysfunction and calcium perturbation induced by traumatic brain injury. J. Neurotrauma 14, 23–34. 10.1089/neu.1997.14.23 9048308

[B61] Youle R. J. van der Bliek A. (2012). Mitochondrial fission, fusion and stress. Science 337, 1062–1065. 10.1126/science.1219855 22936770 PMC4762028

[B62] Yu T. Sheu S. S. Robotham J. L. Yoon Y. (2008a). Mitochondrial fission mediates high glucose-induced cell death through elevated production of reactive oxygen species. Cardiovasc. Res. 79, 341–351. 10.1093/cvr/cvn104 18440987 PMC2646899

[B63] Yu T.-S. Zhang G. Liebl D. J. Kernie S. G. (2008b). Traumatic brain injury-induced hippocampal neurogenesis requires activation of early nestin-expressing progenitors. J. Neurosci. 28, 12901–12912. 10.1523/JNEUROSCI.4629-08.2008 19036984 PMC2605967

[B65] Zhao Y. Chen F. Chen S. Liu X. Cui M. Dong Q. (2013). The Parkinson’s disease-associated gene PINK1 protects neurons from ischemic damage by decreasing mitochondrial translocation of the fission promoter Drp1. J. Neurochem. 127, 711–722. 10.1111/jnc.12340 23772688

[B64] Zhao Y. Cui M. Chen S. Dong Q. Liu X. (2014). Amelioration of ischemic mitochondrial injury and bax-dependent outer membrane permeabilization by Mdivi-1. CNS Neurosci. Ther. 20, 528–538. 10.1111/cns.12266 24712408 PMC6493009

